# Consumers’ Acceptance, Emotions, and Responsiveness to Informational Cues for Air-Fried Catfish (*Ictalurus punctatus)* Skin Chips

**DOI:** 10.3390/foods12071536

**Published:** 2023-04-05

**Authors:** Silvia Murillo, Ryan Ardoin, Witoon Prinyawiwatkul

**Affiliations:** 1 School of Nutrition and Food Sciences, Louisiana State University, Agricultural Center, Baton Rouge, LA 70803, USA; smurillomiguez1@lsu.edu; 2 Food Processing and Sensory Quality Research Unit, Southern Regional Research Center, USDA-ARS, New Orleans, LA 70124, USA

**Keywords:** fish skin, catfish, seafood byproduct, consumer acceptance, food-evoked emotions, purchase intent, product benefits, repeat exposure

## Abstract

Catfish (*Ictalurus punctatus*) skins, as filleting byproduct, were developed into a crispy snack food via air-frying. Consumers rated catfish skin chips (CSC) across sensory modalities (9-point hedonic scales, a just-about-right scale, and “yes/no” for purchase intent, PI) for Plain-, Lemon & Pepper-, and Barbecue-flavored samples during two consumer studies (*N* = 115 each). Paprika- flavored CSC were excluded from Study 2 due to inferior acceptance and emotional ratings. CSC-elicited emotions were evaluated using a 25-term lexicon with CATA (Check-All-That-Apply) scaling (Study 1) and refined with an abbreviated lexicon containing food-evoked sensation-seeking emotions (5-point intensity scale). The two consumer studies differed in delivery format of product benefit information (a health/protein message and a food waste/sustainability message). Presenting two separate cues (Study 1) significantly increased overall liking (by 0.5 units) and PI (by 15%) for CSC compared to a single integrated message (Study 2), perhaps due to consumers’ mode of information processing. Magnitude of increases was less for Barbeque CSC despite performing best overall (overall liking reaching 6.62 and PI reaching 61.7%). CSC generated mostly positive emotions, and informational cues increased sensation-seeking feelings, which can motivate trial of new foods. Accordingly, acceptance of CSC improved for 25 repeat-exposure consumers who participated in both Studies 1 and 2. In combination, sensory, cognitive, and emotional data showed favorable responses for flavored CSC as an appropriate application of this seafood byproduct.

## 1. Introduction

Global fish production (both capture fisheries and aquaculture) is projected to reach 204 million tons by the year 2030, with 89% intended for human consumption [1]. It has been estimated that up to 70% of fish biomass can be wasted during processing, which can have social, economic, and environmental implications [2]. As such, valorization of seafood byproducts has been gaining scientific interest [2,3]. Keeping edible and nutritive fish components, such as skins, in human food systems (rather than waste or non-food applications) can contribute to an increasingly circular and sustainable food supply where natural resources are fully exploited [4]. However, adoption of circular economic principles will require changes in consumption practices [4]. Therefore, consumer insight is needed for effective waste-to-value product development.

Our previous research turned to seafood consumers for indications of product appropriateness for seafood byproducts, which is a cognitive dimension of food choice based on perceived fit or “appropriateness” of an ingredient within a final product concept or preparation [5]. In that study, consumers identified fish products as most appropriate for seafood byproduct incorporation and snack foods as a top-five product category. It was proposed that food made from seafood byproduct could be both a snack and a seafood product unto itself, which motivated the product concept for this consumer research: catfish skin chips (CSC).

Fish skin chips are available commercially, which was verified by a simple internet search, with most resulting products being made from salmon skin. In Asian countries, fried basa fish (*Pangasius bocourti*) skins are marketed as snack food [6]. Research has been conducted on composition, instrumental hardness, and shelf-life of fried tilapia (*Oreochromis* spp.) skin chips [7] and physiochemical and sensory properties of puffed tilapia snacks by air-frying [8] and other cooking methods [6]. While non-traditional cooking methods may lend novelty to these snack preparations, the current research is unique in its development and testing of air-fired chips made from catfish (*Ictalurus punctatus*) skins.

In addition to understanding consumer perceptions of potential new products, investigations of food made from seafood byproduct also present opportunities to explore broader consumer research metrics. Specifically, benefits of food-waste reduction and sustainability, along with nutritional value of fish skin [9], presented the authors with an opportunity to compare formats for product-related informational cue delivery, and their effects on product acceptability and food-evoked emotions in the present research. As an unintended consequence of conducting two sensory studies (*N* = 115 consumers each) at the same location approximately six months apart, we found that twenty-five consumers participated in both studies, thus allowing for a test of repeat-exposure effects on acceptance of CSC.

Previous research has shown that providing consumers with product benefit information after a blind tasting can improve ratings of product liking and purchase intent (PI). This has been demonstrated with informational cues about the fatty acid profile of steaks from grass-fed cattle [10], risks of high sodium intake paired with a “low sodium” claim for roasted peanuts [11], and several other product/benefit combinations. Some studies have integrated multiple pieces of information into a single message, e.g., the satiating, metabolism-boosting, and “good” HDL cholesterol properties of certain oils [12]. Others have presented informational cues separately and sequentially, such as a safety claim for fried fish made with a bone powder breading mix, followed by a separate calcium claim [13]. Still, it is unclear whether multiple pieces of food product benefit information are more impactful when presented in a single integrated format (one cue) or when presented separately (multiple individual cues). The present investigation compared the effects of CSC informational cues in both formats on product acceptability.

The objectives of this research were to investigate consumers’ perceptions of CSC sensory quality with different added flavorings, product-elicited emotions, and effects of informational cues on CSC acceptability and emotions. Additionally, the authors hypothesized that information delivery format (two separate product benefit messages versus one integrated message) would differentially impact consumers’ overall liking and purchase intent of CSC. This was accomplished via two consumer studies at the same location (*N* = 115 each, with 25 repeat participants), which permitted investigation of a repeat-exposure effect on CSC acceptance.

## 2. Materials and Methods

### 2.1. Preparation of Catfish Skin Chips

Fresh channel catfish (*Ictalurus punctatus*) skins (CS) were obtained from a local catfish processor (Breaux Bridge, LA, USA) and transported on ice to the research facility (Baton Rouge, LA, USA). Skins were a byproduct of filleting catfish which yielded 9 to 12 oz (55 cm to 75 cm in length) fillets. Immediately upon arrival at the research facility, the CS were cleaned by passing through water 5 times. CS were then rested on a sieve for 10 min to drain off excess water. CS were dried in an oven (OV310 G rotating rack oven, BAXTER Inc., Deerfield, IL, USA) on aluminum trays for 4 h at 50 °C. Dried CS were left at room temperature overnight to be used the next day for the consumer study. 

On the days of testing, dried CS were cut into squares (5 cm × 5 cm) using only the most uniform part. Square pieces of CS were battered in egg (beaten grade A chicken eggs; Great Value, Walmart, Inc., Bentonville, AR, USA) and coated with flour (all-purpose wheat flour; Great Value). CS squares were air-fried (ten pieces per basket; NINJA^®^ Foodi, SharkNinja Inc., Needham, MA, USA) for 12 min at 199 °C. Preliminary air-frying parameters were considered based on Fang et al. [6], who used 180 °C for up 12 min for tilapia skins. However after in-house evaluations of different conditions, a temperature of 199 °C was found to achieve desired CSC crispiness using the above-mentioned raw material and equipment. Each batch started with a cold unit and took approximately one minute to reach the final set temperature of 199 °C.

Three treatments (flavors) of CSC were prepared by coating air-fired CS with one of the following seasonings (all from McCormick & Company, Baltimore, MD, USA): Lemon & Pepper, LP (salt, black pepper, citric acid, onion, sugar, garlic, calcium stearate, silicon dioxide, calcium silicate, celery seed, lemon oil, and FD&C Yellow N5 Lake), paprika, PK (paprika and silicon dioxide), barbecue seasoning, BBQ (brown sugar, salt, spices including celery, seed, pepper, tomato, garlic, onion, red bell pepper, extractives of paprika, acetic acid, or natural flavor (including hickory smoke)). The plain CSC without any seasoning were considered the control flavor. These flavors were chosen from a range of seasonings based on current snack market trends and pre-screening by ten untrained panelists who ranked the top three seasoned CSC samples based on appearance and flavor. CSC were seasoned, 25 pieces at a time, by shaking them with 60 g of each respective seasoning in a sealed container.

### 2.2. Chemical and Physical Analyses of Catfish Skins and Catfish Skin Chips

#### 2.2.1. Proximate Analysis of Catfish Skins

Proximate analysis and mineral composition analysis of catfish skin were conducted at the Louisiana State University Ag Center Agricultural Chemistry Laboratory (Baton Rouge, LA, USA). Moisture was analyzed following the AOAC Method 934.01, proteins by the AOAC Methods 981.10 and 990.03, fat by AOAC Methods 2003.05 and 2003.07, and ash by AOAC Method 942.05 [14]. 

#### 2.2.2. Color (L*, a*, b*)

Instrumental color was analyzed using a spectrophotometer (CM-5, Konica Minolta Inc., Osaka, Japan). Measurements were recorded as L* (darkness/lightness), a* (greenness/redness), and b* (blueness/yellowness) values. Five measurements per flavor treatment (Plain, Lemon & Pepper, Paprika, and BBQ) were taken. Mean L*, a*, and b* values were used to calculate the delta-E pairwise color differences between treatments (∆E; Equation (1); L*, a* and b* represent means of each respective index, and subscripts _1_ and _2_ refer to two samples of interest [15].

(1) $$\Delta E=\sqrt{{\left(L_{2}^{*}-L_{1}^{*}\right)}^{2}+{\left(a_{2}^{*}-a_{1}^{*}\right)}^{2}+{\left(b_{2}^{*}-b_{1}^{*}\right)}^{2}}$$

#### 2.2.3. Hardness

Hardness of the CSC was measured using a texture analyzer (TA-XTPlus, Texture Technologies, Godalming, UK) equipped with a spherical probe (TA-8, ¼” diameter, aluminum and stainless-steel) and a crisp fracture support rig (Texture Technologies), with a 30 kg load cell. Test parameters were 1 mm/s pre-test speed, 1 mm/s test speed, and 10 mm/s post-test speed. Samples were punctured to a distance of 5 mm. Hardness was expressed as the maximum force recorded during deformation. Ten CSC samples of each flavor treatment (Plain, Lemon & Pepper, Paprika, and BBQ) were measured. 

### 2.3. Consumer Sensory Evaluation

#### 2.3.1. Panelists and Recruitment

Two separate consumer studies were conducted on different days (6 months and 17 days apart), each with a total of 115 consumers; 25 same panelists participated in both studies. Panelists for both studies were recruited from a pool of faculty, staff, and students at the Louisiana State University campus (LSU, Baton Rouge, LA, USA). Participants were at least 18 years old (pregnant women excluded), regular seafood consumers, and had no allergy to fish, wheat, and egg. Informed consent was provided by a signed consent form, and the research protocols were approved by the Louisiana State University Agricultural Center Institutional Review Board (IRBAG-21-0063). Participation was voluntary and no compensation was provided. 

The consumer sensory studies were performed at the LSU AgCenter Sensory Services laboratory (Baton Rouge, LA, USA) in partitioned booths under white light. An online questionnaire (Qualtrics software, Provo, UT, USA) was used to collect data. To avoid bias, samples were labeled with three-digit blinded codes, and unsalted crackers and water were provided for palate cleansing to avoid carryover effects. Microbial analysis was done for CSC samples before the consumer study. APC and E coli/coliforms counts were below the detection level, which was 20 CFU/g and 2 CFU/g, respectively; hence, CSC samples were safe for taste testing.

#### 2.3.2. First Consumer Study (Study 1)

The first consumer sample (*N*_1_ = 115) consisted of 56% males and 44% females. Forty-five percent were US-born (including White, African American, Hispanic, Asian, and other races), and 32% were Hispanic/Latino—not born in the US. As would be expected from sampling on a college campus, consumers were primarily between the ages 18–25 (57%) and 26–35 (30%).

##### Product Acceptability

A total of *N*_1_ = 115 consumers evaluated four CSC samples (Plain, LP, PK, BBQ flavor) which were served following a balanced and randomized complete block design. Panelists were first asked to observe the sample and rate overall visual quality (OVQ) and surface color, then to smell the sample and rate aroma, and finally to taste the sample and rate texture, crispiness, flavor, and overall liking (OL) (all using a labeled 9-point hedonic scale). A 3-point Just About Right (JAR) scale was also used to rate crispiness (Not crispy enough, JAR, Too crispy). Purchase intent (PI) of each sample was reported on a binomial “yes/no” scale. 

##### Informational Cues

After blind (no product information) ratings, two separate product informational cues were provided for each sample. The first cue was a health and protein (HP) message: “This product is a healthy and safe snack prepared in an air fryer (Not using oil) and contains about 16 g (about 50%) of protein per serving size (30 g).” After presentation of the HP message, consumers again rated OL and PI of the sample. 

Then, the second informational cue was presented (a food waste and sustainability (FWS) message): “The consumption of catfish skin will help to reduce food waste and support food sustainability.” Consumers rated OL and PI of the sample a final time. 

##### Emotions

After informational cues, OL, and PI ratings, consumers responded to the question “After visual, aroma and taste evaluations of samples, how does this catfish snack make you feel? Check all that apply.” Consumers selected applicable emotions from a list of 25 terms [16] presented in a randomized check-all-that-apply (CATA) format. These included: active, adventurous, aggressive, bored, calm, disgusted, enthusiastic, free, good, good-natured, guilty, happy, interested, joyful, loving, mild, nostalgic, pleasant, satisfied, tame, understanding, unsafe (related to health), warm, wild, and worried. 

#### 2.3.3. Second Consumer Study (Study 2)

The second consumer sample (*N*_2_ = 115) consisted of 51% males and 49% females. The majority (63%) were US-born (including White, African American, Hispanic, Asian and other races), and 23% were Hispanic/Latino—not born in the US. Consumers were primarily between the ages of 18–25 (66%) and 26–35 (23%).

##### Product Acceptability 

A total of *N*_2_ = 115 consumers evaluated three flavored CSC samples (Plain, LP, and BBQ) which were served following a balanced and randomized complete block design. Paprika flavor was not included in this second study due to the significantly lower acceptance scores compared to the other treatments during the first consumer test. During the second study, consumers rated only OL (on a labeled 9-point hedonic scale) and PI (yes/no scale) of samples. 

##### Informational Cue

After initial OL and PI ratings, a single informational cue was presented, combining both HP and FWS messages. This combined (HPFWS) message informed consumers that “The consumption of catfish skin will help to reduce food waste and support food sustainability. Additionally, this product is a healthy and safe snack prepared in an air fryer (Not using oil) and contains about 16 g (about 50%) of protein per serving size (30 g).” After presentation of the HPFWS message (presented only once in the questionnaire, referring to all samples), OL and PI were rated once more for each sample. 

##### Emotions

Food-evoked emotions were measured at two points during the study using a labeled 5-point Likert-type scale anchored at “strongly disagree” and “strongly agree”. The first instance was immediately after blind ratings of OL and PI: “Please rate each EMOTION term according to your current emotional state after TASTING each sample.” Consumers rated each of eight emotions, which included food-evoked sensation seeking emotions [17] curious, daring, energetic, interested, and wild, as well as negative emotions unsafe, disgusted, and worried presented in a randomized order. These emotions were rated a second time as the last task of the sensory test, after the HPFWS message and final OL and PI ratings. 

### 2.4. Data Analysis 

Mean CSC hardness, color, hedonic scores, and consumer emotions (before HPFSW; Study 2) were compared by analysis of variance (ANOVA). Tukey’s post-hoc test was used for mean separation with physical, chemical, hedonic, and emotion data, and Bonferroni’s adjustment was used with repeated measures ANOVA comparing the effects of separate informational cues on OL during Study 1. Descriptive discriminate analysis (DDA) identified the hedonic attributes influential to overall CSC samples’ differences. A logistic regression model was fit with demographic and hedonic predictors to identify significant predictors of CSC PI for the different informed conditions (before and after HP and FWS messages). McNemar’s test for marginal homogeneity was conducted for significant changes in PI responses with increasing product information. Cochran’s Q test with Bonferroni adjustment compared CATA response frequencies for emotions across CSC treatments. Two-sample t-tests and chi-square tests were used to compare the impact of separate informational cues (HP and FSW) versus the integrated cue (HPFWS) on OL and PI, respectively. Two-sample t-tests and chi-square tests were also used to compare OL and PI, respectively, between repeat-exposure and first-time consumers during Studies 1 and 2. Paired t-tests were used to compare OL of CSC before and after HPFWS messaging and to compare mean emotions ratings (before and after HPFWS; Study 2). Data analysis was conducted using the R software version 4.0.3 (RStudio, Inc., Boston, MA, USA) and Statistical Analysis System (SAS) version 9.4 (Cary, NC, USA).

## 3. Results and Discussion

### 3.1. Proximate Analysis of Catfish Skin

Protein, ash, and fat content of catfish skin in this study were 15.55, 0.37, and 11.63%, respectively (dry weight basis; Supplementary Table S1). These values were slightly lower than results from Bechtel et al., who found 22%, 0.59%, and 14% of protein, ash, and fat, respectively, in channel catfish skin [18]. Higher fat values were attributed to the skinning procedure, in which more of the subcutaneous fat layer may be retained on the skin, and also to the time of the year that samples were collected (late Fall) [18]. However, moisture of the catfish skin was 71%, which was comparable to 65.65% and 69.50% found in previous studies [18,19]. Differences in fish species, habitats, genetics, and diets can have an influence on composition of the skin [20].

### 3.2. Color of Catfish Skin Chips

Color of CSC was variable between treatments within both studies (Table 1), which affected hedonic evaluations. Delta-E values ranged from 3.8 (Plain versus LP) to 13.7 (LP versus Paprika) in Study 1, and from 2.9 (Plain versus LP) to 10.5 (LP versus BBQ) in Study 2. A delta-E color difference above 2.3 has been used as a threshold to indicate noticeable color differences discerned by the human eye [15]. The observed color differences between CSC treatments were expected, as each seasoning had coloring indicative of its intended flavor (e.g., yellow and black particles from LP seasoning). This can be a desirable characteristic of food products, where consumers often use color cues to form flavor expectations, and having that expectation confirmed after tasting leads to more positive experiences than a perceived mismatch between the two modalities [21]. From the present CSC acceptability data, color differences between samples were evident to consumers and proved to be an important factor in discriminating treatments, but relative ratings of color did not directly translate to flavor liking (see Table 2 and Table 3; these results are presented and discussed later). 

### 3.3. Hardness of Catfish Skin Chips

The overall similarity in CSC hardness across treatments prepared at different points in time (no statistical differences between Study 1 and Study 2 samples; Table 1) indicated reproducibility of the CSC production methods, and may also be attributed to consistency of the raw ingredient (catfish skins sourced from a single processor at different times). Other studies have demonstrated that cooking method (e.g., air-frying versus deep frying), time, and temperature can all affect instrumental hardness of fish skin snacks [7,8]. These consistent instrumental CSC hardness results aligned with consumers’ ratings of texture and crispiness liking, discussed in the next section.

### 3.4. Consumers’ Acceptance of Flavored Catfish Skin Chips

Consumer Study 1 (*N*_1_ = 115) collected hedonic information for a range of sensory attributes to identify the most acceptable treatments among Plain, LP, Paprika, and BBQ, beginning with the visual dimension, which is often the first used to evaluate product quality [22]. The most visually acceptable CSC treatment was that flavored with BBQ seasoning (liking of OVQ = 6.30, color liking = 6.47; Table 2), followed by Paprika-flavored treatment (liking of OVQ = 6.27, color liking = 6.25). These samples were similar in the a* and b*color dimensions (higher redness and lower yellowness compared to other treatments; Table 1). For all samples, mean ratings of OVQ (meant to encompass all aspects of product appearance [22]) were similar to those for color (all < 0.2 units apart on the 9-point scale; Table 2).

These results highlight the importance of color as a key aspect of visual quality assessments. In fact, color (pooled within canonical correlation of 0.81, from descriptive discriminant analysis; Table 3), followed by OVQ (0.62), were the two most discriminating hedonic attributes in the first canonical dimension (Can1, which accounted for 70.39% of total variance in sample acceptability; Table 3). Among samples, added seasonings were the only variable expected to influence product appearance. The consistency of CSC color before seasoning was verified by calculating a delta-E color-difference value between the Plain (unseasoned) samples produced for Study 1 versus Study 2. The resultant dealt-E value of 0.97 (calculated from Table 1 data) was indicative of an indiscernible color difference to a normal observer before seasoning was applied [15].

The mean aroma liking of BBQ CSC (6.46 on 9-point scale; Table 2) was also statistically highest among samples (α = 0.05), and BBQ CSC flavor liking was also directionally highest (mean rating of 6.15; Table 2). Aroma and flavor liking scores of samples were moderately correlated (coefficients of 0.73 and 0.64, respectively [23]; Table 3) with variability explained by Can2, which suggests that these attributes were influential in explaining the remaining 29% of variability between samples in the second canonical dimension (Can2, Table 3).

Although flavor accounted for less variability between samples than color and aroma, significant differences were still found. Most notably, flavor of Paprika CSC was least acceptable to consumers and fell into the “dislike” portion of the labeled 9-point scale with a mean liking score of 4.90 (Table 2). Despite similar OVQ and color liking scores between BBQ and Paprika CSC, their relative acceptability diverged after tasting. One potential outcome that arises from a disconfirmation of flavor expectations has been referred to as generalized negativity [23]. This occurs when products are evaluated as inferior due to pre-tasting expectations not being met. Flavor is consistently a predominant sensory driver of food choice, including food made with seafood byproduct [13]. In the present study, flavor liking was the only hedonic dimension to consistently show a significant effect on PI of CSC across all informed conditions (Supplementary Table S2). For these reasons and the negative emotional responses discussed later, the Paprika CSC were not evaluated further in Consumer Study 2.

Both liking of texture and crispiness of CSC were similar across samples of different flavors (all means above 6.2 with no significant differences, Table 2). These results would be expected based on the overall similarities in instrumental hardness (Table 1) and suggested that, overall, consumers were able to properly distinguish textural attributes from other sensory modalities evaluated, including their overall product liking (OL). Crispiness has been defined as both a physical and auditory quality of foods determined during mastication [24]. Crispiness is a characteristic property of snacks such as crackers and potato chips [24] and was sought after in development of the current CSC. Based on logistic regression analysis, a one-unit increase in CSC crispiness liking upon blind tasting would result in an estimated 44% increase in odds of positive purchase intent (odds ratio (OR) = 1.44, Supplementary Table S2). Our results suggested that target crispiness was achieved, as a satisfactory majority of consumers (74.78–80.87% across samples) rated the crispiness of CSC as just-about-right (JAR) (Table 2).

In general, consumers who participated in Study 1 rated the CSC as more acceptable than those from Study 2. Initial OL scores were directionally higher for all CSC flavors in Study 1 than in Study 2, and positive PI proportions were at least as high for each flavor tested (Table 2). The Paprika flavor was excluded from further comparisons because it was not tested in Study 2 due to a mean OL score below 5. Although no significant differences in OL were observed between the other three flavors, BBQ CSC performed numerically best after blind tasting, with mean OL scores of 6.17 (Study 1) and 6.01 (Study 2), both above the “like slightly” category, and positive PI of 49.57% in both studies. These results suggest that appropriate flavor modification can enhance acceptability of CSC, but inappropriate flavor can diminish acceptability.

### 3.5. Effects of Informational Cue Format and Repeat Exposure on Consumers’ Acceptance of Catfish Skin Chips

It was expected that providing consumers with product benefit information related to health (HP) and sustainability (FSW) would directionally improve both OL and positive PI of CSC regardless of format (separate or integrated messages) if consumers’ attitudes toward these concepts were favorable [25]. This was largely the case, as only BBQ CSC decreased in mean OL after the HPFWS message (Study 2), albeit by only 0.03 point on the 9-point scale (Table 4). Positive PI increased for all samples with additional information, significantly so in most instances (Table 2). It was also hypothesized that the format of information delivery (separate versus integrated cues) would affect the magnitude of change in OL and PI. For this analysis, the 115 responses from Study 1 were compared to 90 responses from Study 2, excluding the 25 repeat-exposure consumers who had already received HP and FWS messages.

Separate and sequential informational cues (HP followed by FSW), with OL and PI ratings in between, demonstrated a greater total effect on both OL (mean increase of 0.50 compared to 0.19 units; Table 4) and PI (mean increase of 15.1% compared to 10.7% for “yes” responses) across flavors than did the integrated message (HPFWS). In Study 1, the HP message alone significantly increased OL for all CSC flavors compared to blind tasting (average increase of 0.36 units), and the FWS message significantly increased OL once more. In contrast, the integrated HPFSW message in Study 2 only had a significant effect on OL of Plain CSC (increase of 0.43 units). In both formats, positive PI yielded significant increases based on McNemar’s tests, but the separate messages generated a greater cumulative increase in responses from PI = “no” to “yes” (Table 4).

One possible explanation for the higher observed impact of two separate cues than one integrated cue is the ease of processing simpler messages when making decisions. This may be especially relevant when consumers are not highly motivated, such as volunteer panelists, and may bypass more rigorous information processing in favor of simple heuristics [26]. In the present research, over 20% of Study 1 consumers reported feeling “bored” for all samples (Table 5, discussed later). This phenomenon (called peripheral processing in the elaboration likelihood model of thinking [26]) may have resulted in respondents consolidating the integrated HPFSW message into a single unit of information, whereas presenting HP and FSW separately forced consumers to view each piece of information independently, resulting in an additive effect.

**Figure 1:**
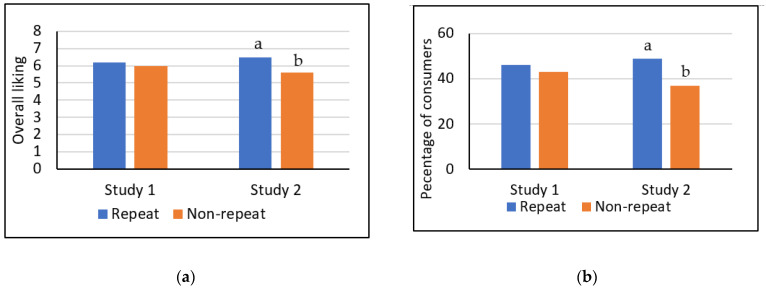
(**a**) Mean overall liking scores (a 9-point hedonic scale) and (**b**) positive purchase intent (PI) for CSC, comparing repeat-exposure consumers (participated in both Study 1 and Study 2; *N* = 25) to those testing CSC for the first time (participated in only Study 1 or Study 2, *N* = 90 for each study). ^a,b^ Different letters indicate a significant difference in OL/PI based on two-sample t-test/chi-square test (α = 0.05).

Consumers’ food choice is influenced by both intrinsic information (e.g., sensory properties) and extrinsic information (e.g., health claims) [23]. It has been suggested that the experienced sensory quality of food (intrinsic) moderates the effect of product benefit information (extrinsic) on subsequent acceptability ratings [13]. Kihlberg et al. [25] found that health information had a greater impact on liking of less acceptable breads than more well-liked samples. A similar effect was noticed in the present study where BBQ CSC performed best in both Studies 1 and 2 upon blind tasting (Table 2) but noticed the smallest increase in PI after informational cues in both formats. The 12.2% total PI increase in Study 1 and 5.6% PI increase in study 2 were both lower than those observed for Plain (15.7% and 16.7%, respectively) and LP (17.4% and 10.0%, respectively) CSC. In Study 2, OL and PI for Plain CSC directionally surpassed those of BBQ CSC. However, it should be noted that, excluding consumers in the repeat-exposure group, acceptability ratings (OL and PI) for CSC were slightly lower overall in Study 2 (Figure 1a,b).

To explore any effect of repeat exposure to CSC on acceptance, we first needed to verify that the 25 consumers who participated in both studies performed similarly to their counterparts upon first exposure (Study 1). These analyses were run excluding the Paprika CSC which were not present during Study 2. A two-sample t-test and chi-square test indicated that OL and PI of CSC for repeat-exposure consumers were not significantly different than of the other 90 consumers who presumably tried the CSC for the first time in Study 1 (mean OL of 6.2 vs. 6.0, and positive PI of 46% vs. 43%; Figure 1a,b). However, after second exposure in Study 2, the repeat-exposure group rated CSC significantly higher in OL than the 90 other first-time CSC consumers (6.5 vs. 5.6) and reported significantly higher positive PI (49% vs. 37%). Therefore, within the current circumstances, a second exposure to CSC improved both OL and PI compared to first-time consumers. However, this hypothesis bears retesting in a planned experiment with a much larger sample size.

### 3.6. Emotional Responses to Catfish Skin Chips

In Study 1, a 25-term emotion lexicon [16] was presented with a CATA scale to allow consumers to identify feelings related to consumption of CSC. King et al. [27] used >20% selection frequency in a CATA format as a criterion to distinguish the most relevant food-evoked emotions. Beyond valence (positive vs. negative), food-evoked emotions have also been categorized by dimensions of arousal (active vs. passive), goal conduciveness (conducive vs. obstructive), and coping potential (high control vs. low control) [17]. Using these criteria, CSC samples were most associated with positive-valence emotions: interested (55–59% selection frequency across CSC flavors), good (45–52%), pleasant (30–40%), adventurous (23–29%), good-natured (21–30%), happy (22–35%), and active (14–28%); as well as low arousal/passive emotions: satisfied (27–50%), calm (26–42%), mild (17–38%), understanding (14–22%), and tame (11–26%), which are also positively valenced (Table 5). Murillo et al. [13] found similar profiles related to catfish strips fried with a bone powder breading mix, indicating generally favorable reactions to consumption of seafood byproducts within a suitable food product.

In the present study, satisfied was the only emotion that significantly differed in selection frequency among treatments; consumers felt less satisfied by Paprika CSC (Table 5). Paprika was the only CSC flavor to yield at least 20% selection of negative emotions worried (20%) and disgusted (25%). These negative reactions to the Paprika CSC further validated the decision to exclude this treatment from further testing in Study 2. Results also demonstrated the efficacy of emotional measurements in discriminating similar products beyond hedonic ratings alone and in guiding product improvement [28]. Bored (17–29% selection rate) was the only negative emotion prevalent among all treatments.

Among the most cited emotions in Study 1 were adventurous and interested, which have recently been coined as food-evoked sensation-seeking emotions (SSE) when related to consumers’ desire for new eating experiences [17]. Therefore, six SSE and three negatively valenced feelings were investigated in Study 2 as related to Plain, LP, and BBQ CSC (Table 6). A mix of negative, positive, and SSE is common in novel food products [29]. Application of a 5-point emotional intensity rating scale was sought to add resolution to measurements beyond CATA scaling. From this abbreviated lexicon, five of the six SSE emerged as significant, either differentiating among CSC flavors or being affected by the HPFWS message.

After receiving HPFSW information, consumers reported feeling more adventurous, daring, interested, and wild. Higher levels of these emotions have been associated with increased willingness to consume new food products [17] and, in the present case, may be indicative of market potential for CSC. Overall CSC evoked higher scores for SSE (2.82–3.75 after HPFWS on a 5-point scale) than in negative emotions (1.89–2.29 after HPFWS). Interestingly, emotion measurement revealed significantly higher states of adventurous and energetic feeling regarding consumption of LP compared to plain CSC. Drivers of consumer food choice are diverse, but by investigating both intrinsic sensory quality and extrinsic product information along with evoked emotions [30,31,32,33], further consideration of BBQ and LP CSC as a viable utilization of catfish skin seems warranted.

## 4. Conclusions

The current methods for producing catfish (*Ictalurus punctatus*) skin chips (CSC) yielded a product that was consistent in color and instrumental hardness, as well as perceived texture and crispiness. This allowed for clear differences in flavor (due to different seasonings) to be revealed, with Lemon & Pepper and Barbecue flavors showing promise to consumers. Consumers responded favorably to product benefit information, more so when health/protein and food waste/sustainability were presented separately rather than as an integrated message. This finding may be useful in communicating the benefits of new products after initial exposure. Upon a second exposure to CSC, overall liking and purchase intent were increased for twenty-five repeat panelists. These results also indicated positive attitudes toward health and sustainability impacts of waste-to-value seafood product development, which increased not only product acceptance but also intensity of food-evoked sensation-seeking emotions and behavioral intent. As the results from analysis of repeat-exposure effects on acceptance and PI of CSC were based on a small sample size, an intended and well-designed study with a much larger sample size is needed to confirm the findings.

## Figures and Tables

**Table 1:** Instrumental color ^1^ and hardness ^2^ values of catfish skin chips.

		Consumer Study 1			Consumer Study 2		
Attribute	Plain	Paprika	Lemon & Pepper	BBQ	Plain	Lemon & Pepper	BBQ
L*	52.3 ± 5.9 ^a^	42.2 ± 0.9 ^b^	52.3 ± 5.9 ^a^	46.3 ± 3.4 ^ab^	52.0 ± 3.2 ^a^	44.9 ± 1.8 ^b^	52.8 ± 4.6 ^a^
a*	4.2 ± 2.2 ^ab^	4.7 ± 1.6 ^a^	1.4 ± 0.7 ^b^	6.5 ± 1.9 ^a^	3.8 ± 2.2 ^ab^	5.7 ± 2.3 ^a^	2.2 ± 0.9 ^b^
b*	10.3 ± 4.4 ^a^	3.7 ± 1.3 ^b^	12.3 ± 1.6 ^a^	5.7 ± 0.7 ^b^	9.5 ± 3.1 ^ab^	5.8 ± 2.3 ^b^	11.8 ± 4.6 ^a^
Hardness (N) ^NS^	13.2 ± 3.0	13.3 ± 3.0	12.2 ± 2.4	12.0 ± 2.7	13.0 ± 3.0	13.3 ± 2.8	13.2 ± 2.6

^1^ Mean ± standard deviation from five measurements. Color was measured as L* (lightness/darkness), a* (greenness/redness), and b* (blueness/yellowness) values. ^2^ Mean ± standard deviation from ten measurements. Hardness (*N* = Newtons) was measured as the maximum force during deformation. ^a,b^ Different letters within a row indicate a significant difference based on ANOVA with Tukey’s post-hoc test (α = 0.05).

**Table 2:** Consumer perceptions of catfish skin from two studies ^1^.

		Flavor ^2^			
	Attribute	Plain	Paprika	Lemon & Pepper	BBQ
Study 1	Overall Visual Quality ^3^	4.98 ± 1.84 ^b^	6.27 ± 1.86 ^a^	5.26 ± 1.86 ^b^	6.30 ± 1.68 ^a^
	Color	4.84 ± 1.90 ^b^	6.25 ± 1.82 ^a^	5.10 ± 1.94 ^b^	6.47 ± 1.67 ^a^
	Aroma	5.50 ± 1.74 ^b^	5.49 ± 1.72 ^b^	6.07 ± 1.86 ^ab^	6.46 ± 1.70 ^a^
	Texture	6.28 ±1.87	6.21 ± 1.91	6.32 ± 1.93	6.39 ± 1.93
	Crispiness	6.78 ± 1.57	6.50 ± 1.79	6.80 ± 1.66	6.63 ± 1.70
	Crispiness JAR (%)	77.39	76.52	80.87	74.78
	Flavor	5.58 ± 1.93 ^ab^	4.90 ± 1.99 ^b^	5.99 ± 2.23 ^a^	6.15 ± 1.96 ^a^
	OL	5.78 ± 1.86 ^abC^	5.29 ± 1.97 ^bC^	6.06 ± 2.01 ^aC^	6.17 ± 1.83 ^aC^
	OL-HP	6.13 ± 1.90 ^abB^	5.68 ± 2.04 ^bB^	6.46 ± 1.99 ^aB^	6.50 ± 1.89 ^aB^
	OL-FWS	6.30 ± 1.92 ^abA^	5.90 ± 2.09 ^bA^	6.59 ± 1.95 ^aA^	6.62 ± 1.82 ^aA^
	PI	40.00 ^B^	32.17 ^C^	40.87 ^C^	49.57 ^B^
	PI-HP	53.04 ^A^	38.26 ^B^	53.04 ^B^	57.39 ^A^
	PI-FWS	55.65 ^A^	44.35 ^A^	58.26 ^A^	61.74 ^A^
Study 2	OL	5.77 ± 1.87	-	5.65 ± 1.74	6.01 ± 1.84
	OL-HPFWS	6.21 ± 2.07	-	5.83 ± 1.79	5.96 ± 1.9
	PI	37.39 ^B^	-	36.52 ^B^	49.57
	PI-HPFWS	54.78 ^A^	-	46.09 ^A^	53.91

^1^ *N* = 115 consumers for each study. Twenty-five same consumers participated in both studies. ^2^ Paprika flavor was excluded from Study 2. ^3^ Overall visual quality, color, aroma, texture, crispiness, flavor, and overall liking (OL) scores are presented as mean ± standard deviation from a 9-point hedonic scale. JAR refers to the percentage of consumers who rated crispiness level as “just-about-right” on a 3-point scale. Purchase intent (PI) is presented as the percentage of “yes” responses. OL and PI were evaluated blind, after a health and protein message (HP), after a food waste and sustainability message (FWS), and after an integrated message (HPFWS). ^a,b,ab^ Values in the same row followed by different lowercase letters are significantly different (ANOVA with Tukey’s post-hoc test; α = 0.05). ^A,B,C^ Values in the same column followed by different capital letters are significantly different (Repeated measures ANOVA for OL/OL-HP/OL-FWS; paired t-test for OL/OL-HPFWS; McNemar’s test for PI/PI-HP/PI-FWS and PI/PI-HPFWS; α = 0.05).

**Table 3:** Descriptive discriminant analysis using liking scores (a 9-point hedonic scale; *N* = 115 consumers) for catfish skin chips.

Variable	Can1	Can2	Can3
	Pooled within Canonical Correlations		
Visual Quality	0.62	0.34	−0.21
Color	0.81	0.36	−0.02
Aroma	−0.01	0.73	−0.41
Texture	−0.02	0.10	0.16
Crispiness	−0.14	0.03	0.01
Flavor	−0.24	0.64	0.45
Overall Liking	−0.19	0.47	0.34
Cumulative Variance Explained (%)	70.39	29.01	0.06
Pr > F	<0.0001	<0.0001	0.9659

**Table 4:** Comparing effects of informational cue format on overall liking and purchase intent (%) of catfish skin chips after each given informational cue.

**Informational Cue (Overall Liking; mean ± SD on a 9-point hedonic scale)**				
**Flavor ^1^**	**HP ^2^**	**FWS ^3^**	**Total ^4^**	**HPFWS ^5^**
Plain	0.36 ± 0.81	0.17 ± 0.62	0.52 ± 0.92	0.43 ± 1.3
BBQ	0.33 ± 0.73 *	0.11 ± 0.84	0.44 ± 1.1 *	−0.03 ± 1.2
LP	0.40 ± 0.78	0.13 ± 0.73	0.53 ± 1.0	0.17 ± 1.6
Across all flavors	0.36 ± 0.77	0.14 ± 0.73	0.50 ± 1.0 *	0.19 ± 1.4
**Informational Cue (Purchase Intent, %)**				
**Flavor**	**HP**	**FWS**	**Total**	**HPFWS**
Plain	13.0	2.6	15.7	16.7
BBQ	7.8	4.3	12.2	5.6
LP	12.2	5.2	17.4	10.0
Across all flavors	11.0	4.1	15.1 *	10.7

^1^ LP = Lemon & Pepper. BBQ = Barbeque. Paprika flavor excluded from comparisons because it was only tested in Study 1. ^2^ Health and protein (HP) message: “This product is a healthy and safe snack prepared in an air fryer (Not using oil) and contains about 16 g (about 50%) of protein per serving size (30 g).” ^3^ Food waste and sustainability (FWS) message: “The consumption of catfish skin will help to reduce food waste and support food sustainability.” ^4^ Total change after both HP and FWS messages, from study 1 (*N*_1_ = 115 consumers). ^5^ A single message containing both HP and FWS information: “The consumption of catfish skin will help to reduce food waste and support food sustainability. Additionally, this product is a healthy and safe snack prepared in an air fryer (Not using oil) and contains about 16 g (about 50%) of protein per serving size (30 g).” From study 2 (*N*_2_ = 90 consumers; repeated exposure consumers from Study 1 excluded). * Significantly different OL/PI than HPFWS based on two-sample t-test/chi-square test (α = 0.05).

**Table 5:** Consumer emotions elicited by catfish skin chip, presented as percentage (%) of *N* = 115 consumers using a check-all-that-apply scale.

Emotion	Plain	Paprika	Lemon & Pepper	BBQ
Active	13.91	15.65	27.83	25.22
Adventurous	23.48	28.70	26.09	27.83
Aggressive	5.22	5.22	7.83	6.96
Bored	23.48	28.70	20.00	16.52
Calm	41.74	33.04	35.65	26.09
Disgusted	14.78	25.22	16.52	15.65
Enthusiastic	10.43	13.91	18.26	19.13
Free	18.26	13.04	16.52	19.13
Good	51.30	45.22	52.17	51.30
Good-natured	24.35	26.96	20.87	29.57
Guilty	4.35	8.70	6.09	6.96
Happy	26.96	22.61	34.78	29.57
Interested	54.78	55.65	54.78	59.13
Joyful	11.30	12.17	20.00	13.91
Loving	4.35	4.35	6.96	6.09
Mild	34.78	38.26	16.52	20.87
Nostalgic	11.30	5.22	9.57	6.09
Pleasant	35.65	29.57	34.78	40.00
Satisfied	35.65 ^a^	26.96 ^b^	33.04 ^b^	49.57 ^a^
Unsafe	4.35	6.96	4.35	6.96
Tame	26.09	25.22	11.30	15.65
Understanding	21.74	13.91	20.00	16.52
Warm	7.83	4.35	15.65	6.96
Wild	6.96	9.57	11.30	7.83
Worried	11.30	20.00	15.65	12.17

^a,b^ Different letters indicate differences in selection frequency (Cochran’s Q test, Bonferroni adjusted *p*-value < 0.0125). Values without superscripts were not significantly different across treatments.

**Table 6:** Emotion intensities (mean ± standard deviation from a 5-point scale) related to catfish skin chips before and after HPFWS ^1^.

Emotion	Before HPFWS			After HPFWS		
	BBQ	LP	Plain	BBQ	LP	Plain
Adventurous	3.44 ± 0.95 ^a^	3.54 ± 1.00 ^a^	2.96 ± 1.06 ^b^	3.69 ± 0.98 ^ef†^	3.75 ± 0.98 ^e†^	3.37 ± 1.17 ^f†^
Curious	3.73 ± 0.97	3.80 ± 0.92	3.5 ± 1.13	3.74 ± 0.95	3.74 ± 1.03	3.61 ± 1.15
Daring	3.13 ± 1.03	3.23 ± 1.06	2.96 ± 1.03	3.38 ± 1.09 ^†^	3.40 ± 1.13 ^†^	3.22 ± 1.11 ^†^
Energetic	3.08 ± 0.95 ^ab^	3.17 ± 1.03 ^a^	2.82 ± 0.96 ^b^	3.16 ± 1.04	3.29 ± 1.01	3.00 ± 1.11
Interested	3.62 ± 1.09	3.57 ± 1.04	3.53 ± 1.16	3.78 ± 0.97 ^†^	3.7 ± 1.01	3.70 ± 1.14 ^†^
Wild	2.85 ± 1.00	2.94 ± 1.05	2.73 ± 1.06	3.09 ± 1.06 ^†^	3.03 ± 1.07	2.88 ± 1.16
Unsafe	1.94 ± 1.06	1.93 ± 0.97	1.89 ± 0.99	2.00 ± 1.06	1.99 ± 1.09	1.91 ± 1.03
Disgusted	2.24 ± 1.10	2.29 ± 1.01	2.18 ± 1.09	2.25 ± 1.19	2.18 ± 1.11	2.11 ± 1.14
Worried	2.11 ± 1.05	2.14 ± 1.06	2.15 ± 1.17	2.03 ± 1.09	2.10 ± 1.14	1.99 ± 1.10

^1^ Emotions were rated before and after a single integrated message containing both HP and FWS information. ^a,b/e,f^ Values in the same row followed by different letters are significantly different. Two ANOVA tests with Tukey’s post-hoc test were run [Before HPFWS] and [After HPFWS] (α = 0.05). ^†^ Significant difference between, before, and after the HPFWS message based on a paired *t*-test.

## Data Availability

The data that support the findings of this study are available from the corresponding author upon reasonable request.

## Supplementary Materials

The following supporting information can be downloaded at: https://www.mdpi.com/article/10.3390/foods12071536/s1, Table S1: Proximate Analysis^ of catfish skin (%, dry wt basis); Table S2: Odds Ratio Estimates^1^ for predicting Purchase Intent (PI) = “yes” based on logistic regression modeling.
