# Supplementary material for: Consumers’ Acceptance, Emotions, and Responsiveness to Informational Cues for Air-Fried Catfish (Ictalurus punctatus) Skin Chips
Source: Foods. 2023 Apr 5;12(7):1536. doi: 10.3390/foods12071536 (PMC10094339; doi:10.3390/foods12071536)
Supplement: Supplementary file 1 [file foods-12-01536-s001.zip › foods-2313894-supplementary.pdf]

foods MDPI

Supplementary Materials

Table S1. Proximate Analysis^ of catfish skin (%, dry wt basis).

Composition	Concentration (%)
Moisture	71.10±0.00
Protein	15.55±0.18
Ash	0.37±0.03
Crude Fat	11.63±0.10

^ Reported as mean ± standard deviation from quadruplications.

Table S2. Odds Ratio Estimates[1] for predicting Purchase Intent (PI) = "yes" based on logistic regression modeling.

Parameters	PIB		PIHP		PIFWS	
	**Pr > ChiSq**	**Odds ratio**	**Pr > ChiSq**	**Odds ratio**	**Pr > ChiSq**	**Odds ratio**
Gender	0.93	0.98	0.78	0.92	0.27	1.35
Nationality	0.28	1.70	0.30	1.86	0.38	0.70
Overall Visual Quality	0.66	0.93	0.92	1.02	0.52	0.91
Color	0.16	1.24	0.87	0.98	0.48	1.11
Aroma	0.66	0.96	0.25	1.13	0.67	1.04
Texture	0.09	1.25	0.03	1.33	0.05	1.27
Crispiness	**0.02**	1.44	0.36	1.14	0.67	1.06
Flavor	**<.0001**	2.69	**<.0001**	2.63	**<.0001**	2.40

[1]Significant odds ratios are in bold typeface (p-value < 0.05). PIB Purchase Intent Before message. PIHP purchase intent after Health and Protein message. PIFWS Purchase Intent after Food Waste and Sustainability message.
